# Chromatin accessibility identifies diversity in mesenchymal stem cells from different tissue origins

**DOI:** 10.1038/s41598-018-36057-0

**Published:** 2018-12-10

**Authors:** Yen-Ting Ho, Takashi Shimbo, Edward Wijaya, Yuya Ouchi, Eiichi Takaki, Ryoma Yamamoto, Yasushi Kikuchi, Yasufumi Kaneda, Katsuto Tamai

**Affiliations:** 1 Department of Stem Cell Therapy Science, Graduate School of Medicine, Osaka University, Suita, Osaka, Japan; 2 StemRIM Co., Ltd., Ibaraki, Osaka, Japan; 3 Division of Gene Therapy Science, Graduate School of Medicine, Osaka University, Suita, Osaka, Japan

## Abstract

Mesenchymal stem cells (MSCs), which can differentiate into tri-lineage (osteoblast, adipocyte, and chondrocyte) and suppress inflammation, are promising tools for regenerative medicine. MSCs are phenotypically diverse based on their tissue origins. However, the mechanisms underlying cell-type-specific gene expression patterns are not fully understood due to the lack of suitable strategy to identify the diversity. In this study, we investigated gene expression programs and chromatin accessibilities of MSCs by whole-transcriptome RNA-seq analysis and an assay for transposase-accessible chromatin using sequencing (ATAC-seq). We isolated MSCs from four tissues (femoral and vertebral bone marrow, adipose tissue, and lung) and analysed their molecular signatures. RNA-seq identified the expression of MSC markers and both RNA-seq and ATAC-seq successfully clustered the MSCs based on their tissue origins. Interestingly, clustering based on tissue origin was more accurate with chromatin accessibility signatures than with transcriptome profiles. Furthermore, we identified transcription factors potentially involved in establishing cell-type specific chromatin structures. Thus, epigenome analysis is useful to analyse MSC identity and can be utilized to characterize these cells for clinical use.

## Introduction

Mesenchymal stem cells (MSCs) have unique differentiation potential toward three mesenchymal lineages including osteoblast, adipocyte, and chondrocyte^1,2^. It was also shown that the differentiation potential of MSCs is not limited to the mesenchymal lineage; specifically, MSCs can also differentiate into ectodermal and endodermal lineages^3^. In addition to their marked differentiation potential, MSCs exert immunosuppressive activity by secreting cytokines such as TSG-6^4^. These unique features make MSCs a promising tool for regenerative medicine for intractable diseases. We previously showed that MSCs have critical roles in tissue regeneration in the damaged skin and can be utilized for the treatment of the intractable genetic skin disease epidermolysis bullosa (EB)^5^. Others have also shown that these cells are potentially useful for various diseases including ischemic stroke and graft-versus host disease^6–9^. Thus, MSCs are anticipated to be effective for treating intractable diseases that require tissue regeneration and immunosuppression.

MSCs are known to have phenotypic diversity^10^. Different factors including tissue origin, gender, age, or culture conditions, can affect the characteristics of these cells^11^. Among these, tissue origin is a key determinant of cell phenotype. MSCs were originally isolated from the bone marrow, but recently it was shown that they can be isolated from multiple tissues such as adipose, lung, umbilical cord, or dental pulp^12–14^. These MSCs with different tissue origins are unique in terms of growth rate, differentiation potential, or cell morphology^15,16^. However, the gold standard to identify the molecular identity of MSCs has not been established. Although cell surface marker analysis using FACS is a standard method to confirm the identity of MSCs, this assay cannot fully account for such diversity, probably because FACS is limited by the number of proteins that it can analyse. Thus, it is essential to uncover the detailed molecular mechanisms that establish MSC diversity.

Recently, the epigenome, a set of information regarding chemical modifications to DNA and DNA-associated proteins, has been extensively analysed to understand the molecular signatures that specify cell identity^17^. Each cell type has a unique epigenome that is used to establish cell-type specific gene expression programs. These cell-type specific gene expression programs are dependent on the well-organized deposition of regulatory proteins such as transcription factors, RNA polymerase, or chromatin remodellers^18,19^. Regulatory proteins dynamically control the 3D structure of the genome and modulate the accessibility of chromatin to establish cell-type specific gene expression programs^20^. To efficiently analyse chromatin accessibility, the assay for transposase-accessible chromatin using sequencing (ATAC-seq) has been recently developed^21^. As ATAC-seq requires fewer cells and handlings compared to conventional techniques, it has been used for various types of cells and has successfully identified their chromatin accessibility profiles^22^.

Here, we describe the comprehensive analysis of MSC signatures to reveal the molecular mechanisms underlying their diversity. Using cells isolated from different tissues as a model to analyse MSC diversity, we simultaneously assessed chromatin accessibility and the transcriptome of MSCs and showed that compared to transcriptome analysis, chromatin accessibility is a superior indicator for cell type identification. We also mapped the regulatory landscape of transcription factors in MSCs to establish cell-type specific gene expression programs.

## Results

### Isolation and validation of MSCs

**Figure 1:**
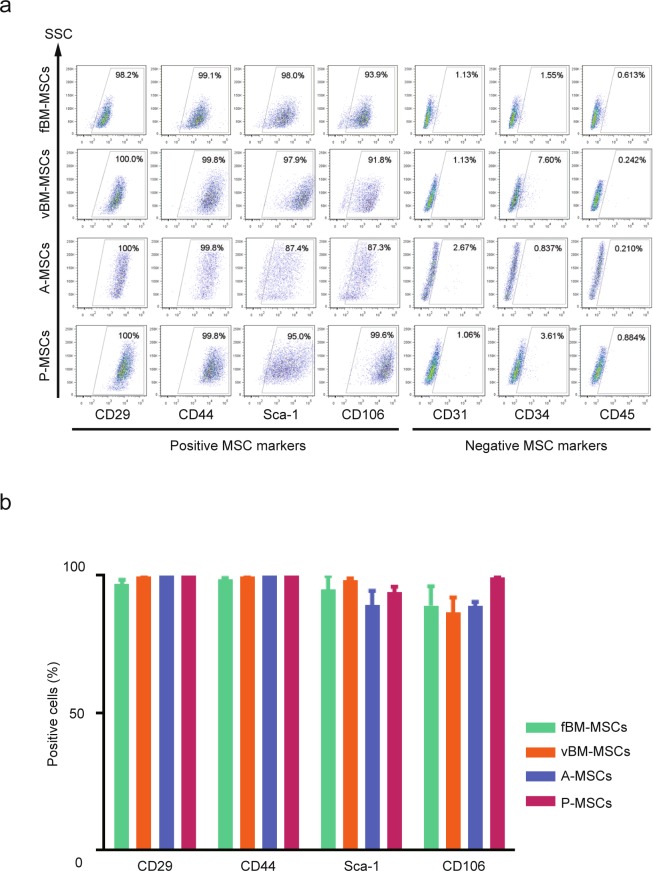
Characterization of cell surface markers on mesenchymal stem cells (MSCs) from different tissue origins. (**a**) Flow cytometric analysis of cell surface markers on isolated MSCs. Positive MSC markers (CD29, CD44, Sca-1, and CD106) and negative MSC markers (CD31, CD34, and CD45) were quantified. Representative results are shown (n = 3). fBM-MSCs, femoral bone marrow MSCs; vBM-MSCs, vertebral bone marrow MSCs; A-MSCs, adipose tissue derived MSCs; P-MSCs, lung derived MSCs. (**b**) Percentages of positive cells for the different MSC markers are shown as mean ± SEM (n = 3).

We first isolated MSCs from four different tissues (femoral bone marrow, vertebral bone marrow, adipose, and pulmonary) using well established protocols (see Experimental Procedures section). We chose these four tissues based on our rationale as follows. In general, the tissue source for MSCs can be categorized as bone marrow or non-bone marrow. First, we chose femoral bone marrow and adipose tissue because these are major sources of bone marrow-type and non-bone marrow MSCs, respectively. To facilitate comparisons within bone marrow type MSCs or non-bone marrow type MSCs, we additionally chose vertebral bone marrow and pulmonary tissue, as the MSCs from these tissues were previously shown to have unique properties^23,24^. Because tissue origin has been implicated in affecting the phenotypes or characteristics of MSCs, we used these cells to model the diversity of MSCs^25^. We collected the four tissues from identical mice and to minimize biological variability, we collected MSCs from three mice (Fig. S1a). To validate the cell isolation and culture protocols, we analysed surface markers on the isolated MSCs by FACS (Fig. 1a and Fig. S1b). All MSCs were positive for MSC makers (CD29, CD44, Sca-1, and CD106) but were negative for non-MSC markers (CD31, CD34, and CD45). In addition, the expression levels of each surface protein were highly consistent among the biological replicates (Fig. 1b). These data confirmed that MSCs were successfully isolated from four different tissues.

### Tissue origin markedly affects MSC transcriptome

**Figure 2:**
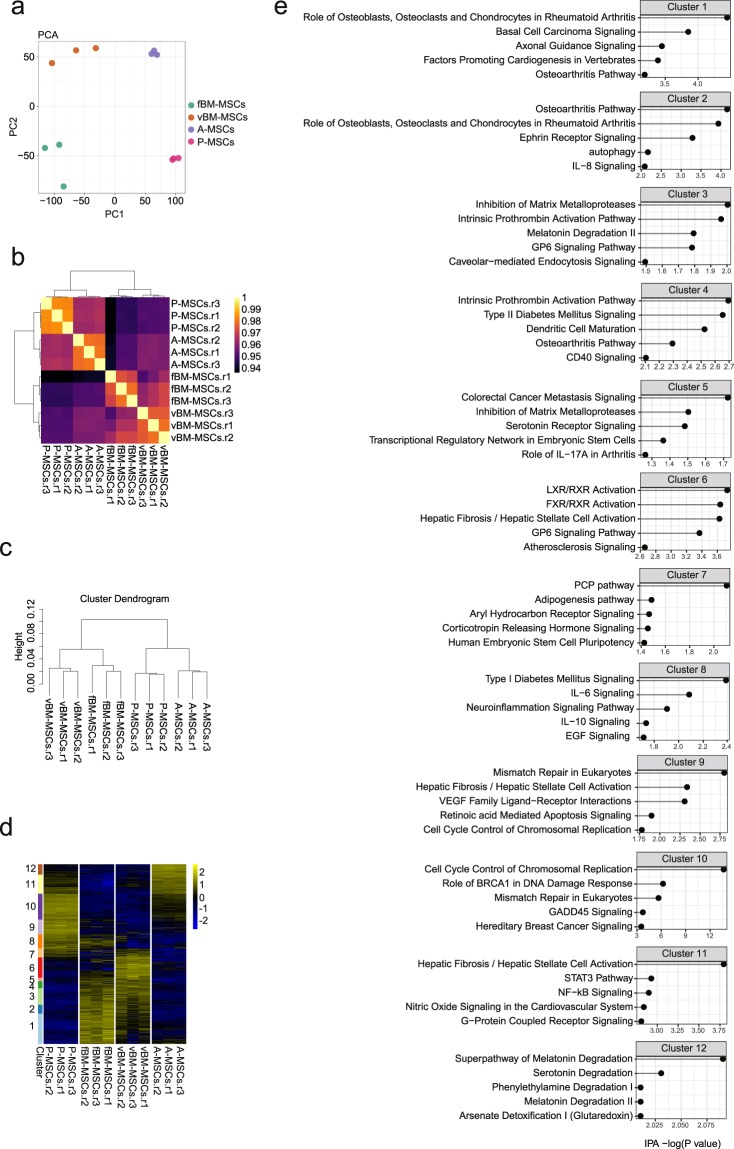
Whole transcriptome analysis of mesenchymal stem cells (MSCs) of different tissue origins. (**a**) Principle Component Analysis using RNA-seq data. Gene expression levels of MSCs were quantified. Each dot represents the gene expression profile of a biological replicate. (**b**) Pearson correlation of RNA-seq data. (**c**) Dendrogram from unsupervised clustering using RNA-seq data. The height indicates the distance between clusters. (**d**) Clustering using an Iterative Clustering and Guide-gene Selection algorithm. The Y-axis indicates sets of genes that were dynamically expressed among cells. (**e**) Pathway analysis of the differentially expressed genes (DEGs) using Ingenuity Pathway Analysis (IPA) software. The genes in each cluster that were dynamically regulated among the mesenchymal stem cells (MSCs) were analysed. Top five canonical pathways identified are shown.

**Figure 3:**
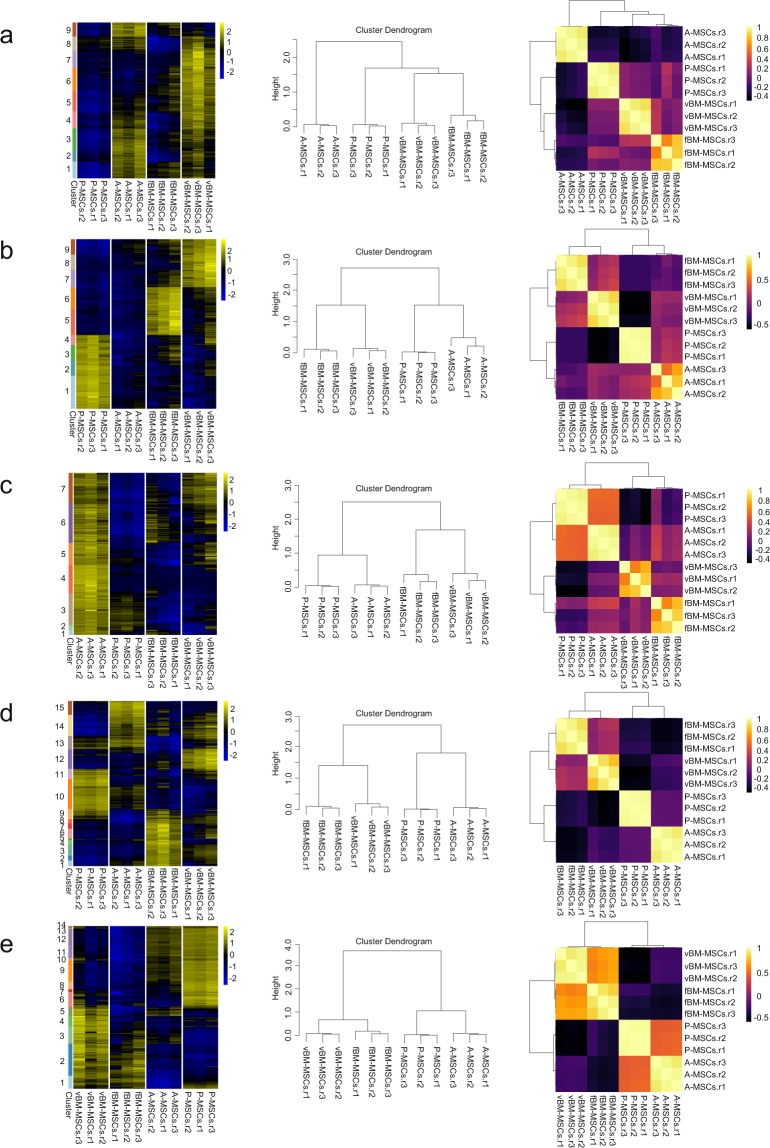
Iterative Clustering and Guide-gene Selection (ICGS) clustering results using different parameters. Threshold for minimum fold-change (FC) in gene expression to detect signature genes: (**a**) FC = 1.5; (**b**) FC = 2; (**c**) FC = 4; (**d**) FC = 5; (**e**) FC = 6.

Next, to further validate the isolated MSCs, we performed whole transcriptome analysis using RNA-sequencing (RNA-seq). We constructed a total of 12 RNA-seq libraries (RNA from the MSCs of four different origins, with three biological replicates for each) using the Smart-seq2 method^26^. First, to visualize differences in the transcriptomes among the four MSC groups, we performed principle component analysis (PCA) using all detected genes (Fig. 2a). The PCA plot clearly separated all four MSC groups, whereas biological replicates from identical tissues clustered together, suggesting that MSC tissue origin is strongly correlated with cell-type specific gene expression patterns. Pearson correlation analysis also confirmed the high correlation between tissue origin and gene expression patterns and confirmed that the biological replicates were in good agreement (Fig. 2b). To further analyse transcriptional differences among the four MSCs, we performed hierarchical clustering (see Experimental Procedures section) and again found that the samples clustered well depending on tissue origin, with all biological replicates clustering together (Fig. 2c). Interestingly, the two bone marrow-derived MSC groups (femoral and vertebral) clustered relatively closely, as compared to the other MSCs, suggesting relatively similar, albeit distinct, transcriptomes between these groups. To further clarify cell type-specific gene expression profiles, we tried to identify the predominant gene expression signatures using the Iterative Clustering and Guide-gene Selection (ICGS) algorithm^27^. ICGS clustered the MSCs based on 12 gene clusters that are uniquely regulated among the cell types (Fig. 2d). To understand the molecular functions of the genes in each cluster, we performed pathway analysis using Ingenuity Pathway Analysis (IPA; Fig. 2e). The pathways related to bone physiology (e.g., role of osteoblasts, osteoclasts, and chondrocytes in rheumatoid arthritis or osteoarthritis pathway) and retinoid X receptor (RXR) activation were predicted for the clusters (cluster 1 to 6) uniquely expressed in bone marrow-derived MSCs (fBM-and vBM-MSCs, femoral bone marrow MSCs and vertebral bone marrow MSCs, respectively). Genes related to mismatch repair were predicted in the lung derived MSCs (P-MSC)-specific cluster (cluster 9) and genes related to melatonin/serotonin degradation were enriched in the adipose tissue derived MSCs (A-MSC)-specific cluster (cluster 12). To investigate how filtering genes into subsets affects the ICGS results, we tried a number of filtering options for RNA-seq using different cut-offs to obtain comparable ATAC-seq clustering results. We found that the filtering could improve the efficiency of clustering using RNA-seq results (Fig. 3). These data indicated that MSCs from different tissues have distinct and unique transcriptomes.

### Chromatin assembly is better at distinguishing MSC origin than transcriptome analysis

**Figure 4:**
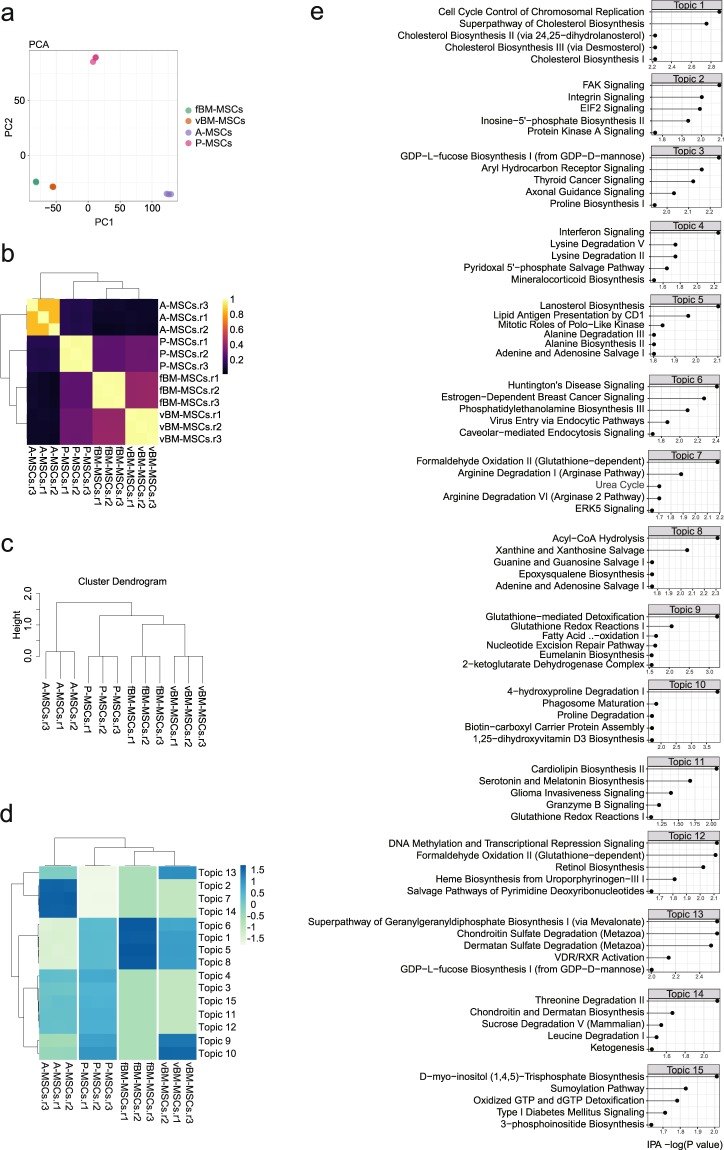
Chromatin accessibility analysis of the mesenchymal stem cells (MSCs) with different tissue origins. (**a**) Principle component analysis using assay for transposase-accessible chromatin using sequencing (ATAC-seq) data from MSCs. Each dot represents the chromatin accessibility profile of a biological replicate. (**b**) Pearson correlation of ATAC-seq data. (**c**) Dendrogram from unsupervised clustering using ATAC-seq data. The height indicates the distance between clusters. (**d**) Clustering using peaks that were dynamically accessible among cells. The variation in chromatin accessibility was assessed by cisTopic. (**e**) Pathway analysis of the differentially accessible regions using Ingenuity Pathway Analysis (IPA). The peaks in each topic that were differentially accessible among the mesenchymal stem cells (MSCs) were assigned to the nearest genes. The resulting gene list was analysed by IPA. The top five canonical pathways identified are shown.

Next, we sought to analyse the epigenomes of the isolated MSCs, which were found to have distinct gene expression profiles. For this, we profiled chromatin accessibility using ATAC-seq. In this technique, sequencing adaptors loaded with Tn5 transposase fragments are used to tag the genome (named “tagmentation”). Because the tagmentation preferentially occurs in the open chromatin regions (relatively protein-free regions), DNA fragments from open chromatin regions can be easily recovered by PCR targeting the adaptor sequence inserted by the transposase. With its robustness and high sensitivity, ATAC-seq has been widely used to profile the epigenome. We constructed 12 ATAC-seq libraries (similar to the RNA-seq experimental design; four different tissues with three biological replicates each). The exemplar locus and basic quality control scores are shown (Fig. S2a). Consistent with other ATAC-seq data, metagene analysis identified the enrichment of ATAC-seq reads that are associated with transcription start sites (Fig. S2b)^28–30^. To obtain information regarding open chromatin regions, we analysed densely-sequenced regions (which represent open chromatin regions) by performing peak call analysis using MACS2. To validate peak calling, we assigned each peak into known genomic features. The majority of called peaks from ATAC-seq data from all four MSCs were located at promoter or genic regions (exons and introns), consistent with other published ATAC-seq data (Fig. S2cd)^28–30^. To further characterize the differences among the MSCs, we selected peaks with high confidence by using the false discovery rate (FDR); peaks with FDR <1e-7 left more than 65000 peaks (approximately 65% of the total peaks) in each group. We then binned the genome using 5-kb sized bins and assigned the read counts to bins where peaks were located. PCA using the assigned score resulted in clear separation of the MSCs depending on tissue origin, whereas biological replicates clustered together, indicating that ATAC-seq data could also predict the identity of MSCs (Fig. 4a). The Pearson correlation heatmap showed significantly low correlations among MSCs from different tissues compared to those based on RNA-seq data, indicating that the differences among MSCs were more pronounced with ATAC-seq data (Fig. 4b). To further confirm this, we performed hierarchical clustering using all ATAC-seq samples and again obtained better separation among tissues compared to that using RNA-seq data, suggesting that ATAC-seq data contains more cell type-specific information to detect the diversity in MSCs (Fig. 4c). In these comparisons, we used RNA-seq and ATAC-seq data without any filtering (except for basic filtering to exclude unreliable signals) to directly compare the nature of the data themselves. To assess whether filtering with RNA-seq data could improve the clustering results, we performed hierarchical clustering using only the differentially expressed genes (DEGs) identified in Fig. 2 and found that RNA-seq could obtain a similar level of clustering power compared to ATAC-seq by performing gene filtering. These data imply that RNA-seq contains features (genes) shared among different cell types compared to ATAC-seq. To predict cell type-specific cis-regulatory programs, we analysed ATAC-seq data using cisTopic^31^. cisTopic identified 15 topics (group of genomic regions that are accessible in a cell type-specific manner) and peaks in each cluster were enriched in unique motifs, suggesting differential molecular mechanisms to regulate each cluster (Fig. 4d). To infer the molecular functions related to each topic, we assigned peaks to the nearest genes and performed pathway analysis (Fig. 4e). Pathway analysis showed unique enrichment of the integrin related pathways (FAK signalling and integrin signalling) in ATAC-seq peaks specific for A-MSCs. Pathways related to cholesterol synthesis (superpathway of cholesterol biosynthesis and lanosterol biosynthesis) and the acyl-CoA hydrolysis pathway were enriched in bone marrow-type MSCs, particularly vBM-MSCs.

### Identification of transcription factors associated with chromatin accessibility among MSC groups

**Figure 5:**
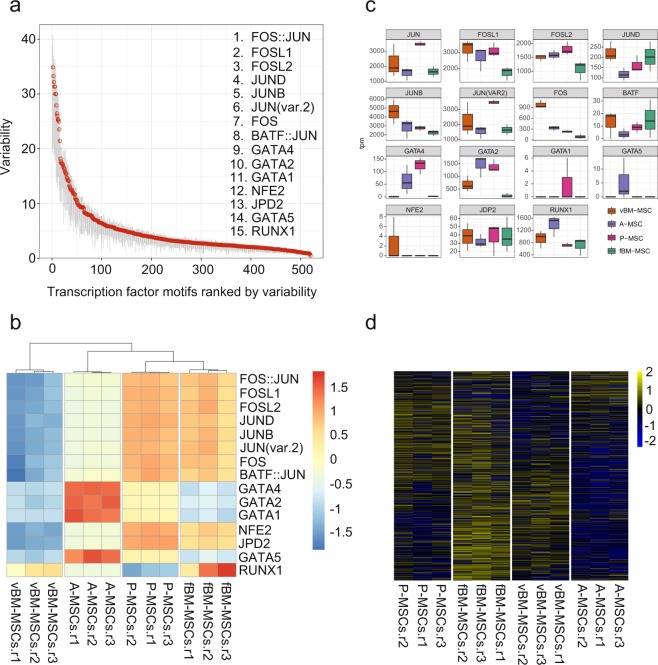
Transcription factor binding motif analyses of mesenchymal stem cells (MSCs) of different tissue origins. (**a**) Motifs with differential accessibility among the MSCs were identified using ChromVar. Identified motifs were sorted using the variability score. (**b**) Motif accessibility in each cell type is visualized using the deviation score. (**c**) Gene expression levels of the transcription factors identified in (**b**). The expression levels were shown as TPM (transcripts per million). (**d**) Association between chromatin accessibility and gene expression. Chromatin accessibility at the promoter regions of the differentially expressed genes (DEGs) identified in Fig. 2d is shown. Promoter region is defined as +/− 1 kb from transcriptional start site (TSS). ATAC-seq read counts in the promoter regions of DEGs were calculated and shown in a heatmap.

To gain a better understanding of the molecular mechanism associated with chromatin accessibility, we searched for transcription factors that are potentially involved in the establishment of cell-type specific chromatin structures in MSCs. To this end, we used chromVAR, which has been specifically developed for ATAC-seq data, to extract transcription factor binding motifs that are accessible in a cell-type specific manner^32^. Using ChromVAR, we identified 414 motifs with differential chromatin accessibility among the MSC groups (p ≤ 0.001) and the top 15 transcription factors are shown in Fig. 5a. To understand the cell-type specific use of the identified transcription factors, we visualized and clustered the accessibility of the top 15 identified variable transcription factor motifs (Fig. 5b). Interestingly, unlike RNA-seq and ATAC-seq, the two MSC groups with a bone marrow origin clustered differently; here femoral bone marrow-derived MSCs and lung MSCs clustered closely. In addition, GATA factors, representing a family known to initiate changes in chromatin structure, were specifically available in adipose tissue-derived MSCs^33^. Similarly, RUNX1, known to be important for haematopoiesis and haematological malignancies, was selectively accessible in femoral bone marrow MSCs^34^. Although most transcription factors identified here were expressed in at least single cell types (except for GATA1 and NFE2 with almost undetectable levels of expression), the expression patterns were not similar to the predicted cell type-specific availability, suggesting that the expression level is not the only determinant factor for the usage of transcription factors in the cells (Fig. 5c). To clarify the functional relationships between RNA-seq and ATAC-seq, we investigated chromatin accessibility at the promoters of the DEGs. The enrichment patterns of the ATAC-seq signals were similar to those for the DEGs, indicating that the DEG promoters were accessible in a manner similar to DEG expression (Fig. 5d).

## Discussion

Here, we demonstrated a strategy to profile the molecular signatures associated with the establishment of the diversity of MSCs. We comprehensively analysed MSCs by RNA-seq and ATAC-seq and found that each group has a distinct transcriptome and epigenome. Our study directly addressed the fundamental question of how each MSC acquires unique features. Applying this strategy to mouse MSCs with different tissue origins identified many known regulators that are potentially involved in the establishment of cell-type specific chromatin structures and gene expression programs.

By comparing RNA-seq and ATAC-seq data without any prior filtering, we found that ATAC-seq contains more information to identify cell type-specific features. As we showed, the expression of cell type-specific genes are well correlated with the chromatin accessibility of the corresponding promoters; further, the gene expression pattern (identified by RNA-seq) and the chromatin accessibility (ATAC-seq) are in a good agreement. In addition to promoter regions, ATAC-seq can identify the open chromatin regions of intergenic regions, which presumably identify potential enhancer regions^22^. Our comparison indicated that this intergenic information is useful to identify cell diversity.

Our data are consistent with previous studies showing a connection between the diversity of MSCs and tissue origin. Although studies addressing the molecular signatures of MSCs at the omics level are limited, Cho *et al*. performed whole transcriptome analysis using human MSCs isolated from bone marrow, adipose tissue, and tonsil and found that MSCs have tissue origin-specific gene expression programs^35^. However, because most studies addressing the diversity of MSCs have used human-derived samples, which inevitably contain variations caused by genetic and/or environmental factors, it was difficult to precisely evaluate the influence of tissue origin on MSC diversity. In this study, by choosing a mouse model to control sample-to-sample variation, we successfully concluded that the diversity of MSCs is influenced by tissue origin. In addition, we analysed cultured MSCs, rather than primary MSCs, in this study because we aimed to characterize cultured MSCs that are being used for many pre-clinical and clinical studies^36^. However, although the isolation of primary MSCs is challenging due to the lack of suitable cell surface markers and the paucity of MSCs in the body, it would be of interest to perform similar analyses using primary MSCs^37^.

In this study, we used the Smart-seq2 protocol, which was developed for RNA-seq using small amounts of RNA (minimum requirement is approximately 10 pg of RNA), to perform whole transcriptome analysis. Although Smart-seq2 is a very powerful method to analyse the full-length transcriptome, there are a few limitations associated with this technology, as compared to other RNA-seq methods. First, because Smart-seq2 is dependent on oligo dT primers to convert RNA into cDNA, it can only analyse polyadenylated RNA and not non-polyadenylated RNA such as histone mRNAs, long non-coding RNAs, nascent RNAs, and enhancer RNAs^38^. In addition, the use of oligo dT primers, instead of random hexamers, can be biased towards the 3′ end of transcripts. Second, Smart-seq2 is not a strand-specific method. Further studies with multiple RNA-seq methods would be helpful to comprehensively compare the transcriptomes and epigenomes of MSCs.

Our initial efforts to characterize the molecular signatures of MSCs using ATAC -seq have led to the identification of potential transcription factors that might be useful to distinguish MSCs with different characteristics. We speculate that this approach, namely profiling MSCs by focusing on chromatin accessibility, could also by useful to characterize human MSCs. Considering the clinical use of human MSCs, it is important to fully characterize these cells to assure treatment safety and efficacy. Profiling MSCs using ATAC-seq might help to standardize preparation protocols for clinical use.

## Methods

### Mice

C57BL6/J mice (8-week-old) were purchased from CLEA Japan (Tokyo). All mice were housed under a 12-h light-dark cycle and were provided solid food and filtered water. All animals were handled in accordance with the approved guidelines of the Animal Committee of Osaka University Graduate School of Medicine. The protocols were approved by the Animal Committee of the Osaka University Graduate School of Medicine.

### Isolation and culture of MSCs from tissues

Mouse bone marrow-derived MSCs were isolated from the bone marrow of the femur and vertebrae by crushing the bone with a mortar and pestle in MesenCult medium (STEM CELL technologies)^39^. For the isolation of lung and adipose-derived MSCs, tissues were minced into pieces and digested with MesenCult medium containing 0.2% collagenase (Wako) at 37 °C for 30 min^13,40^. The collagenase was removed by washing twice with 1× PBS. The cell suspension was filtered through a cell strainer (40-μm) and collected in a 50-ml tube. Red blood cells were removed by incubating cells in 1× RBC lysis buffer (BioLegend) for 5 min at room temperature. Then, 2 × 10^7^ cells were seeded onto a collagen I-coated, 10-cm dish using MesenCult medium containing 1× MesenPure and 10 nM of a Rock inhibitor. MSCs were cultured for up to three passages for experiments. All MSCs were used within three passages to reduce any artefacts potentially introduced by long-term culture^41^.

### Flow cytometric analysis

One million cells were aliquoted in 100 μl of cell staining buffer (PBS with 2% FBS) and incubated with purified rat anti-mouse CD16/32 antibody for 10 min at 4 °C, followed by the fluorophore-conjugated antibodies of cell surface markers for 20 min at 4 °C as follows: FITC anti-mouse/rat CD29 (clone HMβ1-1, BioLegend), PE anti-mouse CD31 (clone 390, BioLegend), PE anti-mouse CD34 (clone MEC14.7, BioLegend), PE anti-mouse/human CD44 (clone IM7,BioLegend), PE anti-mouse CD45 (clone 30-F11, BioLegend), PE anti-mouse Sca-1 (clone D7, BioLegend), PE anti-mouse CD106 (clone 429, BioLegend), and PE anti-mouse IgG (isotype control) antibodies. Stained cells were analysed using the FACSCantoII system (BD Bioscience).

### RNA-seq library preparation

RNA was extracted using the RNeasy Plus Micro kit (QIAGEN) and RNA concentration was determined using a Qubit 3.0 Fluorometer (Thermo Fisher Scientific). RNA quality was assessed on an Agilent 4200 tapestation instrument (Agilent Technologies). RNA-seq libraries were constructed following the Smart-seq2 protocol^26^.

### ATAC-seq

ATAC-seq libraries were constructed in accordance with a previously published protocol with minor modifications. Tagmentation was conducted with the Nextera DNA Library Prep kit (Illumina). Five thousand cells were resuspended in 50 μl of transposase mixture (25 μl of 2× TD buffer, 2.5 μl of TDE1, 1 μl of 5% IGEPAL-CA600, and 22.5 μl of nuclease-free water) at 37 °C for 30 min. DNA was extracted using the Zymo clean & concentrator kit (Zymo research). Transposed DNA was combined with the PCR mix (5 μl of 25 μM Nextera PCR primer, 25 μl of NEB High Fidelity 2× PCR master mix, 10 μl of nuclease-free water) and amplified using the following conditions: 72 °C for 5 min; 98 °C for 30 s; five cycles of amplification at 98 °C for 10 s, 63 °C for 30 s and 72 °C for 1 min. To enrich for transposed DNA of optimal sizes (150 to 500 bp), dual-size selection was performed using AMpure XP beads (BeckmanCoulter). Size-selected transposed DNA was combined with PCR mix (0.5 μl of 25 μM Nextera PCR primer, 0.06 μl of 100× SYBR green1, 7.5 μl of NEB High Fidelity 2×PCR master mix, 1.94 μl of nuclease-free water) and amplified by performing additional PCR cycles determined by qPCR, which was followed by purification with AMPure XP beads. The concentration of purified DNA was determined using the Qubit 3.0 Fluorometer (Thermo Fisher Scientific) and DNA size was analysed with a Bioanalyzer High sensitivity DNA chip (Agilent Technologies).

### Analysis of RNA-Seq data

The mouse reference genome (mm10) was obtained from the iGenomes repository (https://support.illumina.com/sequencing/sequencing_software/igenome.html.). Fastq data for RNA-seq were aligned using RSEM with the following parameters (rsem-calculate-expression –paired-end –star –output-genome-bam)^42^. The clustering procedure is based on two unsupervised methods. First, the hierarchical clustering method was made with the R *heatmap.2* function using ward.D2 and the Pearson correlation as a measure of distance. Second, PCA-based clustering were processed using the R function *prcomp* with default parameters. These steps were applied to all genes as features, with TPM-based expression. The analyses of differential gene expression were performed using AltAnalyze using the following parameters (*–runICGS yes –column_method hopach –rho 0.4 –SamplesDiffering 3 –excludeCellCycle conservative –ExpressionCutoff 4 –FoldDiff* 3)^27^. In Fig. 3 we show the results with *FoldDiff*: 1.5, 2, 4, 5, 6.

We applied IPA for pathway analysis of the DEGs focusing on canonical pathways. In Fig. 2e we highlight the top five pathways ranked according the −*log(Pvalue)* of IPAs.

### ATAC-Seq data analysis

Fastq data for ATAC-seq data were processed using TrimGalore (https://github.com/FelixKrueger/TrimGalore) with the following parameters (–stringency 5 –paired –trim1 –length 30 -q 0 –a CTGTCTCTTATACACATCT) to trim adaptor sequences and then aligned using Bowtie^43^ with the following parameters (-m 1 -v 2 -S -I 0 -X 2000). Duplicated reads were removed using Picard’s MarkDuplicates (http://broadinstitute.github.io/picard/) with default settings. To account for the Tn5 transposon cleavage position in the mapped reads, we shifted the mapped reads by +4/−5 bp depending on the strand. The reads mapped to the blacklist features as defined in the ENCODE project were removed^44^. Peak calling was performed using MACS2 with the following parameters (macs2 callpeak –nomodel –nolambda –keep-dup all –call-summits)^45^. Based on this output, we created the bigWig file using UCSC bedGraphToBigwig to visually inspect the data. For clustering and correlation analysis, we filtered the significant peaks (FDR <1e-7) from the MACS2 output peak list and then the read density within a peak was calculated using featureCounts^46^. We conducted quantile normalization and GC content correction using the R package CQN^47^. For further downstream analysis, we binned the genome into 5-kbp windows and assigned the normalized read density to the corresponding bins (the peaks that spanned more than two windows were reassigned according the ratio of the base pair overlap). All feature intersections were done using BEDTools^48^. Peak annotation to given genomic features was performed using Homer^49^. Transcription factor motif analyses were performed using chromVar^32^ with JASPAR’s vertebrates’ motif set^50^. We analysed the differentially accessible regions in ATAC-seq data (Fig. 4d) using cisTopic^31^. We explored six possibilities of models (2, 5, 10, 15, 20, 25). Our analysis showed that the topic with 15 models was most suitable with the lowest log-likelihood. The pathway analysis of these topics (Fig. S5) was performed using IPA, wherein the genes were obtained from the TSS region that overlapped with topic region (BED) within +/− 1 kbp distance. The genes of the corresponding transcription factors were derived from RNA-seq data processed according to the description of the previous section (Fig. 5c). We constructed the heatmap (Fig. 5d) by first obtaining the TSS region (+/− 1 kbp) of corresponding DEGs and performed the read counting using ATAC-seq data. We further performed the GC correction on these read counts with CQN^47^.

### Statistical analysis

Statistical analyses were performed using Prism7 (GraphPad software). Data are shown as the mean ± S.E.M and statistical significance was defined at *P < 0.05 based on a one-way ANOVA followed by a Turkey’s multiple comparison test.

### Accession numbers

All sequencing data reported here are deposited at GEO and available under the Accession Number GSE116558.

## Electronic supplementary material

Supplementary info
