# Supplementary material for: Chromatin accessibility identifies diversity in mesenchymal stem cells from different tissue origins
Source: Sci Rep. 2018 Dec 10;8:17765. doi: 10.1038/s41598-018-36057-0 (PMC6288149; doi:10.1038/s41598-018-36057-0)
Supplement: Supplementary file 1 — Supplementary info [file 41598_2018_36057_MOESM1_ESM.pdf]

Chromatin accessibility identifies diversity in mesenchymal stem cells from different tissue origins

Yen-Ting Ho, Takashi Shimbo, Edward Wijaya, Yuya Ouchi, Eiichi Takaki, Ryoma Yamamoto, Yasushi Kikuchi, Yasufumi Kaneda, Katsuto Tamai

Supplementary Figure S1

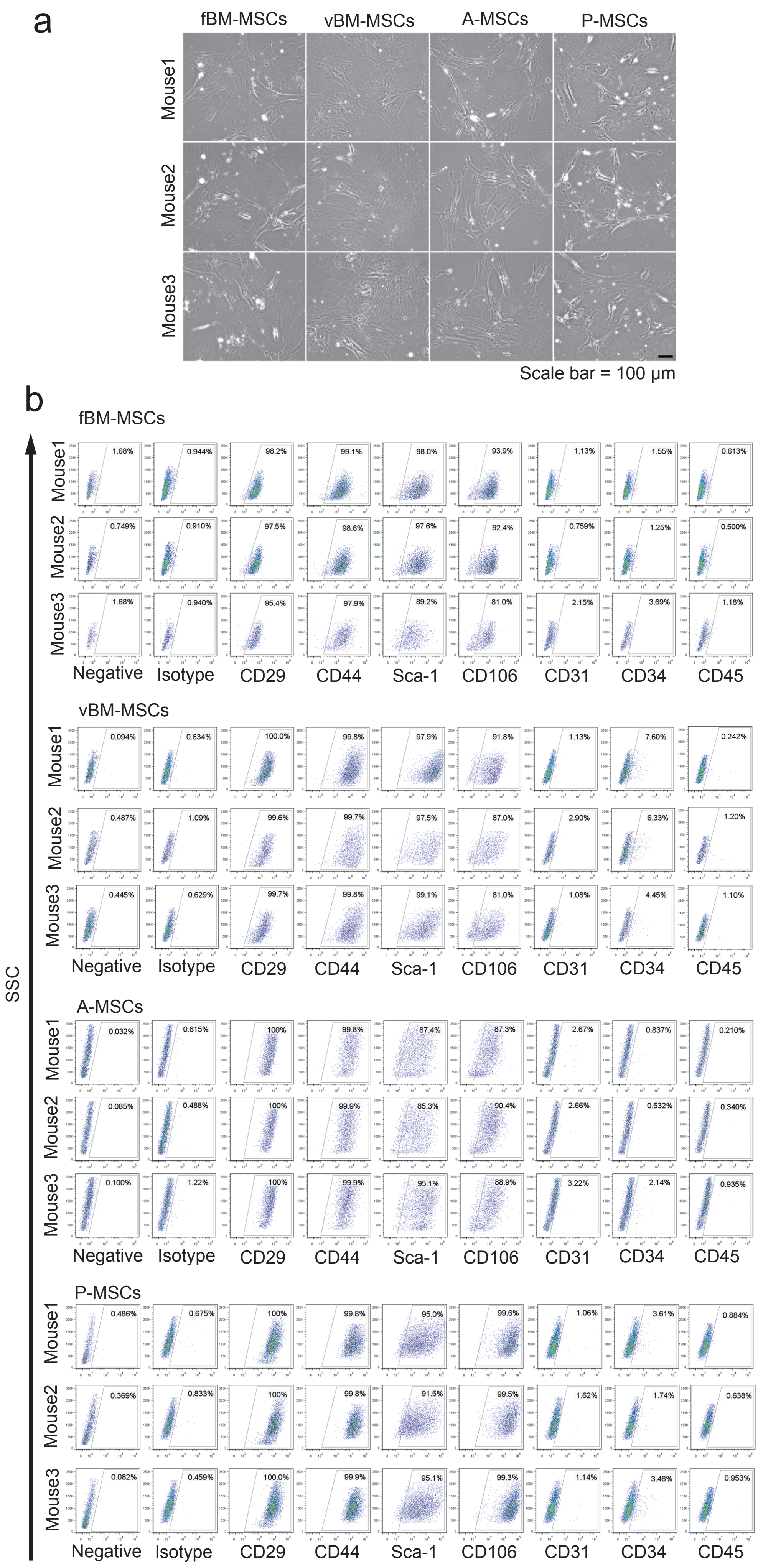

Supplementary Figure S2

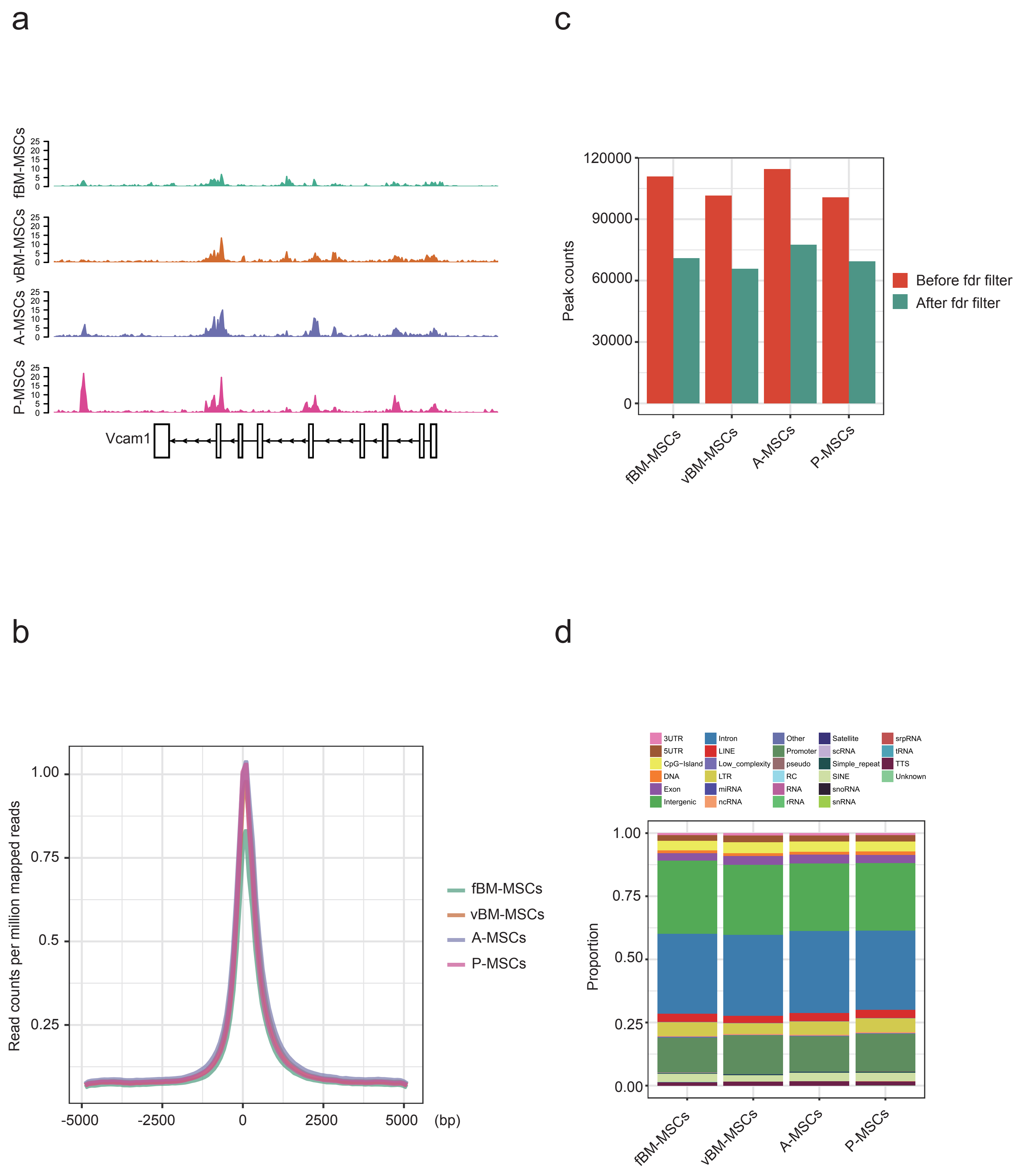

Supplementary Figure legends

Figure S1. Isolation of mesenchymal stem cells (MSCs) from four different tissues. (a) Representative images of each MSC replicate. (b) All FACS results corresponding to Figure 1.

Figure S2. Assay for transposase-accessible chromatin using sequencing (ATAC-seq) on mesenchymal stem cells (MSCs). (a) ATAC-seq was performed on MSCs derived from different tissue sources. An exemplar genome browser depiction of an ATAC-seq result around the Vcam1 gene. (b) ATAC-seq read counts at promoter regions are shown. TSS, transcription start site. (c) Number of ATAC-seq peaks called using MACS2. Peaks were filtered using FDR. (d) Each peak was assigned genomic features.
